# High Basal Expression of Interferon-Stimulated Genes in Human Bronchial Epithelial (BEAS-2B) Cells Contributes to Influenza A Virus Resistance

**DOI:** 10.1371/journal.pone.0109023

**Published:** 2014-10-14

**Authors:** Lai-Giea Seng, Janet Daly, Kin-Chow Chang, Suresh V. Kuchipudi

**Affiliations:** School of Veterinary Medicine and Science, University of Nottingham, Sutton Bonington Campus, Leicestershire, United Kingdom; University of Regensburg, Germany

## Abstract

Respiratory epithelial cells play a key role in influenza A virus (IAV) pathogenesis and host innate response. Transformed human respiratory cell lines are widely used in the study of IAV−host interactions due to their relative convenience, and inherent difficulties in working with primary cells. Transformed cells, however, may have altered susceptibility to virus infection. Proper characterization of different respiratory cell types in their responses to IAV infection is therefore needed to ensure that the cell line chosen will provide results that are of relevance *in vivo*. We compared replication kinetics of human H1N1 (A/USSR/77) IAVs in normal primary human bronchial epithelial (NHBE) and two commonly used respiratory epithelial cell lines namely BEAS-2B and A549 cells. We found that IAV replication was distinctly poor in BEAS-2B cells in comparison with NHBE, A549 and Madin-Darby canine kidney (MDCK) cells. IAV resistance in BEAS-2B cells was accompanied by an activated antiviral state with high basal expression of interferon (IFN) regulatory factor-7 (*IRF-7*), stimulator of IFN genes (*STING*) and IFN stimulated genes (ISGs). Treatment of BEAS-2B cells with a pan-Janus-activated-kinase (JAK) inhibitor decreased IRF-7 and ISG expression and resulted in increased IAV replication. Therefore, the use of highly resistant BEAS-2B cells in IAV infection may not reflect the cytopathogenicity of IAV in human epithelial cells *in vivo*.

## Introduction

Influenza A viruses (IAVs) infect a wide spectrum of avian and mammalian hosts with a range of clinical manifestations from mild or in-apparent infection to severe fatal disease. The dangerous features of IAVs are their highly infectious nature, broad host range, and their inherent nature to frequently mutate and/or reassort from mixed infections, resulting in emergence of new strains. Despite ongoing vaccination programmes, influenza is still one of the leading causes of human deaths, with 3–5 million cases of severe illness and up to 500,000 deaths each year (http://www.who.int/mediacentre/factsheets/fs211/en/). IAVs have been shown to readily overcome vaccine specificity and develop resistance against the few available antiviral drugs such as neuraminidase inhibitors [1].

While seasonal influenza virus infection typically causes contagious respiratory disease in humans with relatively low mortality rate, infections with some of the highly pathogenic avian influenza (HPAI) viruses of the H5N1 subtype could cause up to 60% mortality [2]. The unusual severity of HPAI H5N1 virus infections in humans has been attributed to hyperacute dysregulation of pro-inflammatory cytokines and chemokines often referred to as a ‘cytokine storm’ [3], [4]. It has previously been shown that pigs are inherently more resistant to HPAI H5N1 virus infection than humans, with reduced infectious virus production and pro-inflammatory response [5]. It is increasingly clear that host responses critically contribute to the disparate clinical outcomes of IAV infection.

Respiratory epithelial cells are the primary early targets of IAVs and are key players in the host innate immune responses to IAV infection. The first step in IAV infection is the attachment of viral haemagglutinin (HA) to host cell sialic acid (SA) receptors [6]. Avian influenza viruses show preferential binding to SA receptors linked to galactose with an alpha 2–3 linkage (SAα 2,3 Gal) whereas classical human influenza viruses preferentially bind to alpha 2,6 linked SA receptors (SAα 2,6 Gal) [7], [8]. Human respiratory epithelial cells express both alpha 2,3 and alpha 2,6 receptors [9] and are permissive to a range of avian and mammalian influenza virus replication. Hence, respiratory epithelial cells are important in the study of influenza virus−host interactions.

As alternatives to primary normal human bronchial epithelial (NHBE) cells, immortalised human respiratory cell lines are often used as they are easily accessible, cost-effective and can be propagated almost indefinitely. For example, adenovirus-12 SV40 transformed human bronchial epithelial cells (BEAS-2B) and adenocarcinomic human alveolar basal epithelial cells (A549) are two of the most common cell lines used in the study of IAV−host interactions [10]–[12] and in infections with other respiratory viruses such as human respiratory syncytial virus [13], [14].

Many immortalized cell lines such as Madin-Darby canine kidney (MDCK), Vero and baby hamster kidney (BHK21) cells are readily permissive to influenza virus replication and are also widely used for *in vitro* virus infection studies. However, IAV replication kinetics can vary depending on the virus strain and the type of cell line. For example, MDCK cells support more rapid growth of influenza viruses than Vero cells [15]. However little is known about the differences in IAV replication between primary human respiratory epithelial cells and cell lines. It has been shown that antiviral response pathways are dysregulated in cancer cells due to immortal transformation [16]–[18]. Therefore, there may be differences in antiviral mechanisms between primary cells and transformed cells that could result in differences in virus replication and cellular responses to virus infection.

Consequently, it is likely that IAV replication kinetics and cellular responses to virus infection could be different between primary and immortalized respiratory cells. Proper characterization, in particular of viral growth, in different respiratory cell types is therefore needed to allow rational selection of the most appropriate cells for addressing specific influenza research questions.

In order to characterize differences in virus replication between human primary and transformed respiratory epithelial cells, we compared virus replication and cellular responses to human H1N1 IAV infections in NHBE, BEAS-2B and A549 cells. We found that BEAS-2B cells are highly resistant to avian and human IAV infections in comparison with NHBE and A549 cells.

## Materials and Methods

### Cells and viruses

BEAS-2B (Sigma Aldrich) and NHBE (Lonza) cells were cultured in bronchial epithelial growth medium (BEGM, Lonza) at 37°C in an atmosphere of 5% CO_2_. A549 cells (ATCC CCL-185) and MDCK cells were cultured in Dulbecco's Modified Eagle's Medium (DMEM) supplemented with 100 units/ml penicillin and 100 µg/ml streptomycin (Invitrogen), 10% fetal bovine serum (FBS) and 0.3 g/l L-glutamine. A549 cells were switched to BEGM 48 h before virus challenge.

A low pathogenicity avian influenza (LPAI) H2N3 virus (A/mallard duck/England/7277/06) and a moderately pathogenic human influenza H1N1 (A/USSR/77) virus were used. All viruses were grown by allantoic inoculation of 10-day-old embryonated hens' eggs. Viruses were titrated in MDCK cells using an immunocytochemical focus assay [19].

### Virus infection of cells

At 80% confluence, cells were rinsed twice with phosphate buffered saline (PBS) and infected with H1N1 or H2N3 IAVs at multiplicity of infection (MOI) of 1.0, based on virus titration values on MDCK cells, in infection medium comprising 2% Ultroser G (Pall Biosepra, Portsmouth, UK), 500 ng/ml TPCK trypsin (Sigma-Aldrich Ltd.) and antibiotics in Ham's F12. At 2 h incubation, cells were rinsed twice with PBS and fresh infection medium added. Cells were further incubated for 4, 6 or 22 h. Cells infected for 6 h were fixed in acetone: methanol (1∶1) for 10 min and were subjected to immunocytochemical staining using a murine monoclonal antibody to influenza nucleoprotein (NP) as previously described [5]. At 10 and 24 h post infection, culture supernatants were collected for infectious virus titration on MDCK cells as previously described [19]. Total RNA was extracted using RNeasy plus kit (Qiagen) following the manufacturer's instructions.

### Influenza receptor detection

Influenza virus receptors on cultured cells were characterized using FITC-labelled *Sambucus nigra agglutinin* (*SNA*) (Vector Labs) for SAα 2,6 Gal, and biotinylated *Maackia amurensis* agglutinin II (*MAA* II) (Vector Labs) for SAα 2,3 Gal in a previously described lectin-cytochemical method [20].

### Influenza PB1 protein expression

Infected cells were lysed using RIPA lysis buffer (Santa Cruz) and cellular proteins were separated on a Tris-glycine gel and blotted onto polyvinylidene difluoride (PVDF) membrane. Viral polymerase basic 1(PB1) protein expression was detected by western blot analysis using a goat polyclonal primary anti-PB1 antibody (Santa Cruz), followed by donkey anti-goat IgG-horseradish peroxidase (IgG-HRP) (Santa Cruz), and subsequently visualized by standard enhanced chemiluminescence reaction ECL detection kit (Amersham Life Science Ltd).

### Viral and host gene expression

Quantification of expression of viral and host genes based on cDNA converted from total RNA (Superscript III first strand cDNA synthesis kit, Invitrogen) was performed on a LightCycler-96 (Roche, Mannheim, Germany) using the SYBR green or TaqMan method. Primers and probe used for detecting influenza matrix (M) gene expression were as previously described [21]. Primers for the expression analysis of *IL-6, IFNβ, and Mx1* were as described in Nelli et al. (2012) [22]. Predesigned primers (KiCqStart SYBR Green Primers) for expression analysis of *ISG15*, *STING*, *IRF-7* and *IRF-3* were purchased from Sigma Aldrich. Other primer sequences are as follows: interferon beta (*IFN-β*) sense: 5′- ACCTCCGAAACTGAAGATCTCCTA-3′, antisense: 5′- TGCTGGTTGAAGAATGCTTGA-3′; *ISG15* sense: 5′- AGATCACCCAGAAGATCG-3′, antisense: 5′-TGTTATTCCTCACCAGGATG-3′; *STING* sense: 5′-CTATTTCTACTACTCCCTCCC-3′antisense: 5′- CGCAGATATCCGATGTAATATG-3′; *Mx1* sense: 5′- TTCTGGGTCGGAGGCTACAG-3′, antisense: 5′- TGGATGGCGGCGTTCT-3′; *IRF-7* sense: 5′- TCTTCTTCCAAGAGCTGG-3′, antisense: 5′- CTATCCAGGGAAGACACAC-3′; *IL-6* sense: 5′- GGTACATCCTCGACGGCATCT-3′, antisense: 5′- GTGCCTCTTTGCTGCTTTCAC-3′. Data were normalized to 18S rRNA expression using a relative standard curve method [23].

### JAK inhibition

BEAS-2B cells were treated with 2.5 or 5.0 µM concentration of pyridine 6 (P6) [2-(1,1-dimethylethyl)-9-fluoro-3,6-dihydro-7H-benz[h]-imidaz[4,5-f]isoquinolin-7-one, Calbiochem], a pan-Janus-activated-kinase (JAK) inhibitor [24]. JAK inhibitor P6 was dissolved in DMSO. Following pre-treatment of cells with different concentrations of P6 or corresponding amounts of DMSO as controls for 24 h, medium was removed and cells were rinsed with PBS before infecting with H1N1 influenza virus at MOI of 1.0, based on viral titration values on MDCK cells. Cells were immunostained for viral NP at 6 h of infection. At 24 h infection, ISG expression, influenza viral M gene was quantified from total RNA, and progeny virus output in culture media was determined by immunocytochemical focus assays on MDCK cells.

### Statistical analysis

Statistical analyses for quantitative reverse transcriptionPCR (qRT-PCR) data were carried out using Relative Expression Software Tool (REST, Qiagen) [25]. Statistical analysis of infectious virus titration data was performed by a two-sample *t* test using Minitab software version 16 (16.2.2.).

## Results

### BEAS-2B cells were highly resistant to influenza virus infection relative to MDCK cells

**Figure 1:**
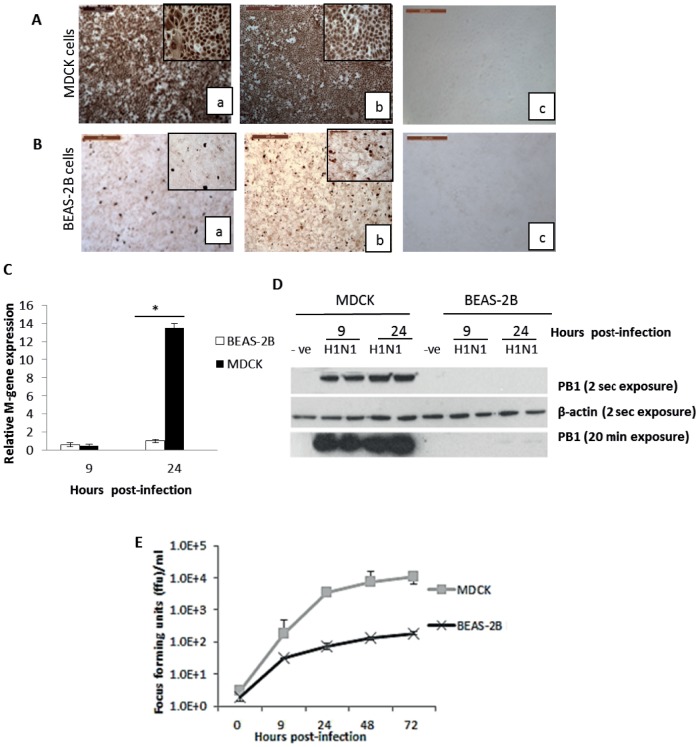
BEAS-2B cells were considerably less permissive to influenza virus replication than MDCK cells. (A) MDCK and BEAS-2B cells were infected with USSR H1N1 or H2N3 influenza viruses at 1.0 MOI (based on MDCK cells), and viral NP expression was determined at 6 h post-infection (hpi). All MDCK cells were positive for viral NP at 6 hpi with (Aa) human H1N1 and (Ab) avian H2N3 viruses. (B) In contrast, few corresponding virus positive BEAS-2B cells were found (Ba and Bb). Mock infected MDCK (Ac) and BEAS-2B (Bc) cells showed no staining. MDCK cells and BEAS-2B cells were infected with H1N1 virus (dose equivalent to MOI 1.0 as per MDCK titration) to determine cellular expression of viral M gene RNA, normalized to 18Sr RNA (C), PB1 protein expression at 9 and 24 hpi (D), and infectious virus output at 9, 24, 48 and 72 hpi (D). In BEAS-2B cells, (C) viral M gene expression was much lower than MDCK cells, (D) little or no PB1 protein was detected (only detected faint bands for PB1 after prolonged exposure for 20 min) and (E) less infectious progeny virus was produced over 72 h of infection. ** (p<0.01).

BEAS-2B and MDCK cells were infected with USSR H1N1 and LPAI H2N3 IAVs at MOI of 1.0, based on viral titration values on MDCK cells. MDCK cells infected for 6 h with human H1N1 (Figure 1Aa) or avian H2N3 (Figure 1Ab) IAVs showed 100% detection of viral NP. In sharp contrast, less than 5% of BEAS-2B cells were positive for viral NP (Figure 1B).

To evaluate the kinetics of virus replication, cellular expression of viral M gene RNA, viral PB1 protein and infectious virus production were compared between H1N1 virus-infected MDCK and BEAS-2B cells. At 24 h infection, M gene expression was significantly higher (p<0.05) in MDCK cells than in BEAS-2B cells, which showed little increase in M gene expression with time (Figure 1C).

Protein expression of viral PB1 protein was barely detectable in H1N1 virus infected BEAS-2B cells whereas corresponding MDCK cells consistently showed much higher levels of PB1 (Figure 1D). Similarly, BEAS-2B cells released significantly less (p<0.05) infectious virus than MDCK cells at 9, 24, 48 and 72 h of infection (Figure 1E).

### BEAS-2B and MDCK cells showed similar distribution of human and avian influenza virus receptors

**Figure 2:**
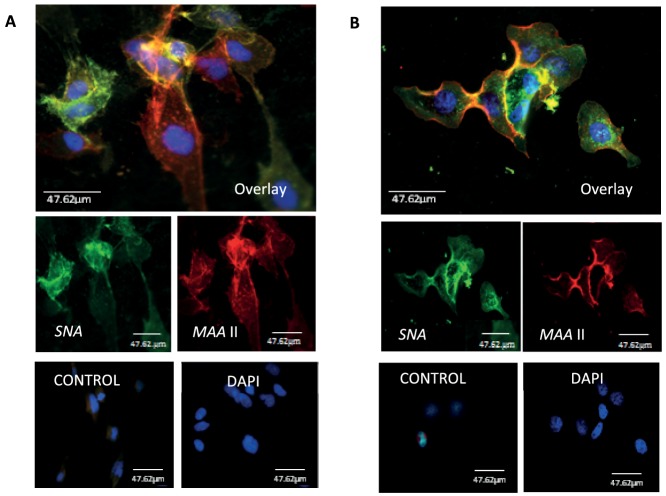
Similar distribution of human and avian influenza virus receptors on BEAS-2B and MDCK cells. Human (SAα2,6-Gal [green]) and avian (SAα2,3-Gal [red]) virus receptors were detected on (A) BEAS-2B and (B) MDCK cells by lectins *SNA* and *MAA* II, respectively. Co-expression of both receptors was found in BEAS-2B and MDCK cells. Negative controls were performed without lectin incubation.

To determine if the cellular distribution of virus receptors on BEAS-2B and MDCK cells could account for the difference in viral replication between the two cell types, SA-linkage specific lectin cytochemistry was performed. Similar co-expression of both human (SAα2,6-Gal [green]) and avian (SAα2,3-Gal [red]) type influenza virus receptors was found in BEAS-2B (Figure 2A) and MDCK cells (Figure 2B), suggesting that there was no qualitative difference in virus receptors between the two cell types.

### BEAS-2B cells were more resistant than human primary NHBE cells and human A549 cells to human H1N1 and avian H2N3 influenza viruses

**Figure 3:**
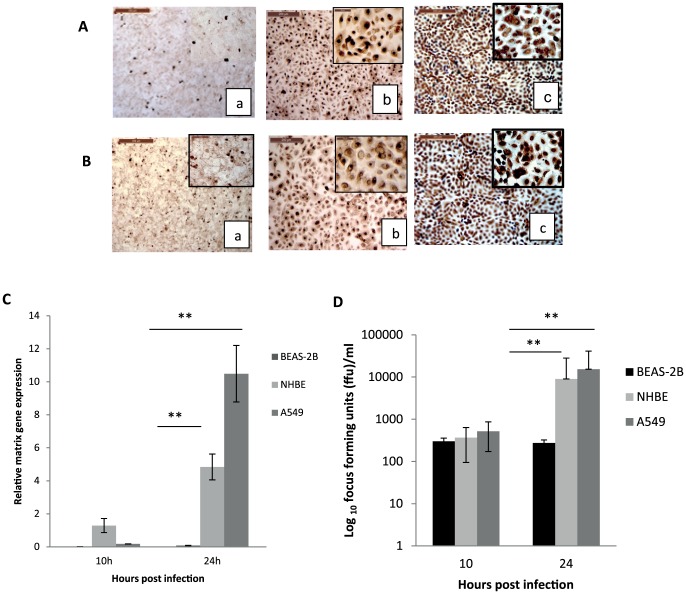
BEAS-2B cells were more resistant than human primary NHBE cells and human A549 cells to human H1N1 (A) and avian H2N3 (B) influenza virus. Cells were infected with the respective virus at a dose that was equivalent to 1.0 MOI in MDCK cells. For both viruses, few BEAS-2B cells (a), most NHBE cells (b), and virtually all A549 cells (c) were positive for viral NP at 6 h of infection. At 24 h of infection with human H1N1 virus (C and D), M gene expression (C) and infectious virus production (D) from BEAS-2B cells were significantly less than corresponding NHBE and A549 cells, with A549 cells showing the highest M gene expression (C) and infectious virus production (D). **p<0.01.

To compare the susceptibility of BEAS-2B cells with primary NHBE and A549 cells, the three cell types were infected with USSR H1N1 or avian H2N3 viruses at MOI of 1.0 (based on MDCK cells). Viral NP at 6 h of infection with H1N1 virus (Figure 3A) and H2N3 virus (Figure 3B) showed that few BEAS-2B cells, most NHBE cells and virtually all A549 cells were positive for viral NP. We then quantified viral M gene RNA expression and progeny virus release in all three cell type at 10 and 24 h of H1N1 virus infection. M gene expression (Figure 3C) and infectious virus production (Figure 3D) from BEAS-2B cells were significantly less than corresponding NHBE and A549 cells at 24 h of H1N1 virus infection (p<0.05), with A549 cells showing the highest M gene expression (Figure 3C) and infectious virus production (Figure 3D).

### BEAS-2B cells displayed differential accumulation of human H1N1 and avian H2N3 viruses in extended cultures

**Figure 4:**
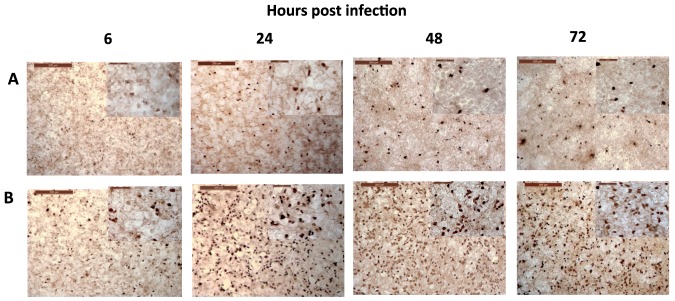
BEAS-2B cells showed greater accumulation of avian H2N3 virus than human H1N1 virus in prolonged cultures. At 6h of infection with H1N1 (A) and H2N3 (B) virus (at MOI 1.0 based on MDCK cells titration), there was little difference in virus accumulation as determined by viral NP detection. However, over 72h of H2N3 virus infection, there was progressive increase in NP intensity and number of infected cells (B). Such increases were barely detected in H1N1 virus infected BEAS-2B cells (A).

As BEAS-2B cells were more resistant to influenza virus infection (Figures 1 and 3), they were infected with human H1N1 and avian H2N3 viruses (at 1.0 MOI based on MDCK cells) over an extended period of 72 h (Figure 4). At 6 h of infection, there was little difference in virus accumulation as determined by viral NP detection. However over 72 h of H2N3 virus infection, there was progressive increase in NP intensity and number of infected cells (Figure 4B). Such changes were barely detected in H1N1 virus infected BEAS-2B cells (Figure 4A). Thus, virus accumulation in resistant BEAS-2B cells varied according to virus subtype.

### BEAS-2B cells showed high basal expression of *IRF-7* and several ISGs

**Figure 5:**
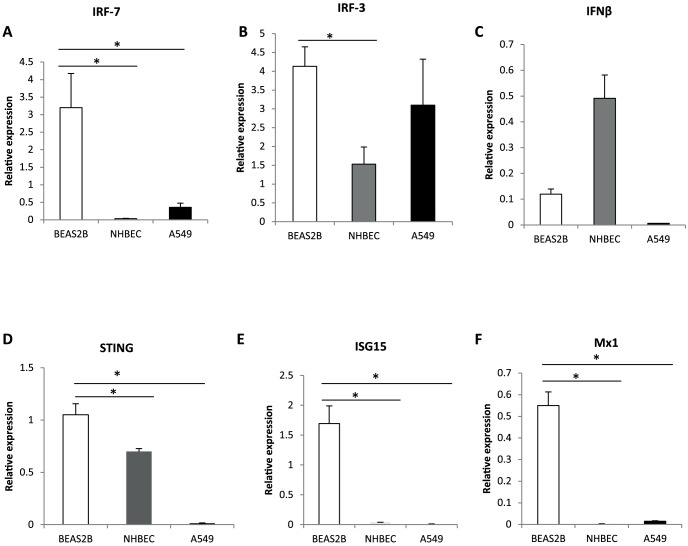
Resistance of BEAS-2B to influenza virus infection associated with high basal expression of *IRF-7* and several ISGs. The basal expression of a selection of genes associated with antiviral function was determined in the three human epithelial cell types (BEAS-2B, NHBE and A549 cells). In BEAS-2B cells, (A) basal *IRF-7* expression was significantly higher than the other two cell types (p<0.05). Basal basal expression of (D) *STING* (E) *ISG15* and (F) *Mx1* in BEAS-2B cells was also significantly higher (p<0.05) than in NHBE and A549 cells. Data normalized to 18S rRNA expression. Data points represent mean of 3 biological replicates. Error bar  =  SEM, *p<0.05.

To gain insight into the relative resistance of BEAS-2B cells to H1N1 influenza virus infection, the basal expression of a selection of genes associated with antiviral function was determined in the three human epithelial cell types (BEAS-2B, NHBE and A549 cells) (Figure 5). In BEAS-2B cells, basal *IRF-7* expression was significantly higher than the other two cell types (p<0.05) (Figure 5A). Notably, basal expression of *STING* (Figure 5D), *ISG15* (Figure 5E) and *Mx1* (Figure 5F) in BEAS-2B cells was also significantly higher (p<0.05) than in NHBE and A549 cells.

### BEAS-2B cells displayed strong induction of *IRF-7* and ISGs at 24 h of H1N1 virus infection

**Figure 6:**
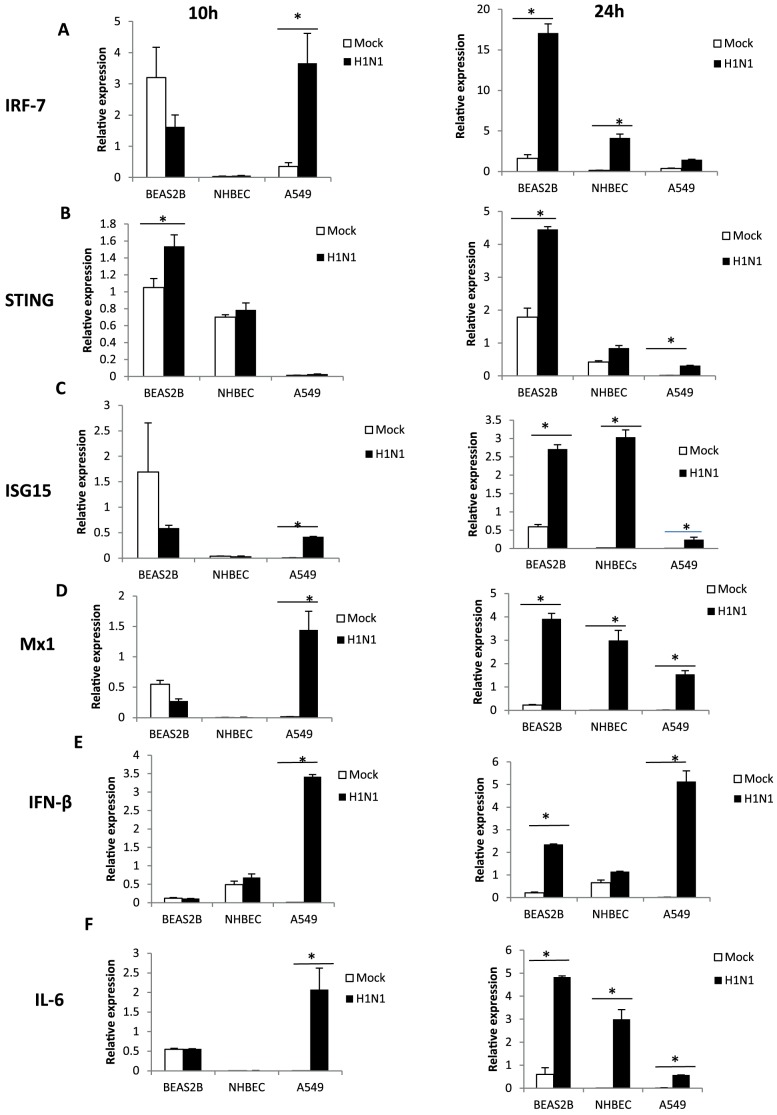
BEAS-2B cells displayed strong induction of *IRF-7* and several ISGs at 24 h of H1N1 virus infection. Human epithelial BEAS-2B, NHBE and A549 cells were infected with human H1N1 virus at 1.0 MOI (based on MDCK cells). At 10 h of infection, expression pattern of antiviral genes (A) *IRF-7*, (B) *STING* (C) *ISG15*, (D) *Mx1*, (E) *IFN-β* and (F) *IL-6* was unremarkable in BEAS-2B and NHBE cells; however *IRF-7*, *ISG15*, *Mx1*, *IFN-β* and *IL-6* expression were up-regulated in A549 cells. Notably, all genes analyzed in BEAS-2B cells were significantly up-regulated by 24 h of infection; BEAS-2B cells expressed the highest *IRF-7*, *STING*, *Mx1* and *IL-6* RNA amongst the three cell types. Data normalized to 18S rRNA expression. Data points represent mean of 3 biological replicates. Error bar  =  SEM, *p<0.05.

To evaluate the antiviral response to influenza virus, the three human epithelial cell types were infected with the human H1N1 virus at 1.0 MOI (based on MDCK cells). In BEAS-2B cells at 10 h of infection, *IRF-7* (Figure 6A), *ISG15* (Figure 6C) and *Mx1* (Figure 6D) expression were significantly down-regulated (p<0.05) while *STING* expression (Figure 6B) was significantly up-regulated (p<0.05); expression of *IFN-β* and *IL-6* was unchanged (Figure 6 E–F). NHBE cells showed no significant change in antiviral gene response, and in *IFN-β* and *IL-6* expression at 10 h infection (Figure 6E–F). In corresponding A549 cells, all antiviral genes (except *STING*), *IFN-β* and *IL-6* were significantly up-regulated (p<0.05) (Figure 6A–F). By 24 h of infection, however, expression of all four anti-viral genes and *IFN-β* and *IL-6* was clearly up-regulated (p<0.05) in all three cell types; BEAS-2B cells displayed the highest expression of *IRF-7*, *STING*, *Mx1* and *IL-6*.

### JAK-STAT pathway inhibition increased susceptibility of BEAS-2B cells to influenza virus infection

**Figure 7:**
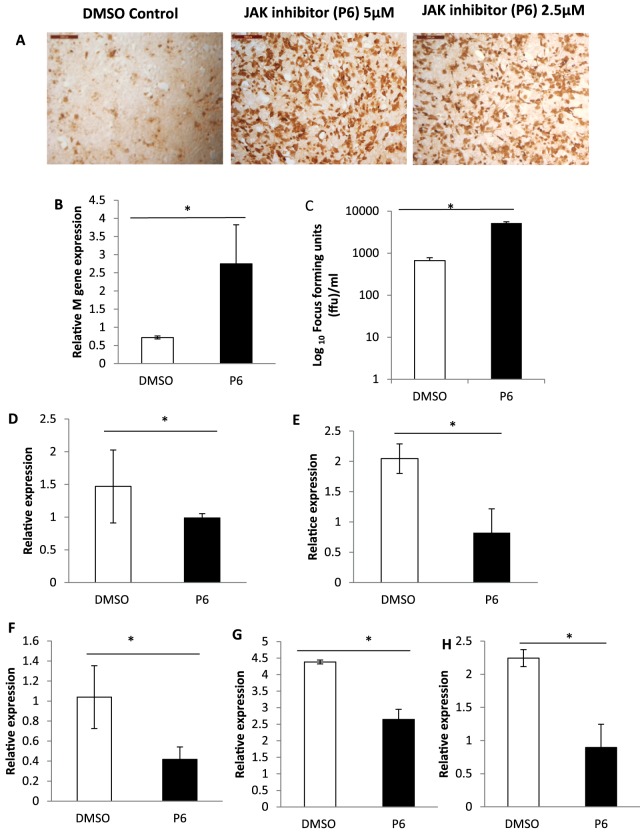
Pan-JAK inhibitor P6 decreased ISG expression and increased susceptibility of BEAS-2B cells to H1N1 influenza virus infection. (A) BEAS-2B cells treated with P6 inhibitor showed enhanced susceptibility to H1N1 influenza virus infection as evident by greater viral NP expression in P6 treated cells compared with DMSO control. BEAS-2B cells were pre-treated with P6 at a final concentration of 2.5 or 5 µM for 24 h and infected with H1N1 influenza virus at 1.0 MOI (based on MDCK cells). At 6 h post-infection cells were immunostained for viral NP. (B) P6 treated (2.5 µM) BEAS-2B cells showed higher influenza M gene RNA expression and (C) produced higher levels of infectious progeny virus at 24 h of H1N1 influenza virus infection. Increased influenza virus replication in P6 treated BEAS-2B cells corresponded to decreased expression of (D) *IRF-7* (E) IL-6 (F) *ISG15* (G) *STING* (H) *Mx1* compared with the controls. Total RNA extracted at 24 h infection from P6 or DMSO pre-treated cells was used for gene expression analysis re normalized to 18S rRNA expression. Culture supernatants collected at 24 h virus infection from P6 or DMSO pre-treated cells were subjected to infectious virus titration on MDCK cells using an immunocytochemical focus assay. Data points represent mean of 3 biological replicates with error bars showing standard deviation, *p<0.05.

To assess the role of high basal and strong induced expression of ISGs at 24 h of influenza virus infection, BEAS-2B cells were treated with pyridine 6 (P6), a pan-JAK inhibitor known to shut down ISG expression [24]. P6-treated BEAS-2B cells showed increased susceptibility to H1N1 virus infection as seen by greater number viral NP positive cells at 6 h of infection compared with DMSO controls (Figure 7A). BEAS-2B cells treated with P6 (2.5 µM) showed significantly higher (p<0.05) M gene RNA (Figure 7B) and produced significantly (p<0.05) higher levels of infectious progeny virus at 24 h of virus infection (Figure 7C). As expected the expression of anti-viral genes namely IRF*-7*, *IL-6*, *ISG15*, *STING* and *Mx1* was significantly reduced in P6 treated BEAS-2B cells.

## Discussion

In this study, BEAS-2B cells were shown to be significantly resistant to avian H2N3 and human H1N1 IAV infection compared with MDCK, NHBE and A549 cells. Influenza virus PB1 protein is an essential subunit of influenza virus RNA polymerase and plays a key role in influenza virus replication [26]. Poor replication of influenza viruses in BEAS-2B cells was associated with little PB1 protein expression in contrast to corresponding abundance of PB1 in MDCK cells. Viral HA binding to host SA receptors initiates IAV replication and the specific SA receptor distribution pattern of cells determines IAV susceptibility [20]. We found similar patterns of co-expression of avian and mammalian type influenza receptors in BEAS-2B and MDCK cells, which indicated that the resistance of BEAS-2B cells was not due to the absence of specific virus receptors.

Comparison of the kinetics of IAV replication showed that BEAS-2B cells were also highly resistant to IAV replication compared with the NHBE and A549 cells. In contrast to our findings, an earlier study found similar viral PB1 gene expression between BEAS-2B and A549 cells [27]. The reason for this difference is not clear, however the previous finding used different influenza virus strains, i.e. A/Udorn/72 (H3N2) and A/WSN/33 (H1N1), and the results were based on qRT-PCR quantification of PB1 gene expression rather than PB1 protein expression and infectious virus release. Thus it is possible that the choice of virus strains or a difference in the methodology may be responsible for the different observations.

Influenza virus replication in cell cultures can be different depending on the origin of influenza viruses. For example, human, avian and swine influenza viruses showed different replication kinetics in NHBE cells with human IAVs growing to higher titres [28]. In contrast, we found that BEAS-2B cells were relatively more resistant to human H1N1 than avian H2N3 influenza virus infection. These findings suggest that BEAS-2B cells do not reflect the natural host restriction pattern of NHBE cells to IAV infection.

In order to establish the molecular basis for the IAV resistance in BEAS-2B cells, we evaluated the regulation of antiviral pathways before and after H1N1 virus infection. We found significantly higher basal expression of *IRF-7* in BEAS-2B cells compared with NHBE and A549 cells. *IRF-7* plays a key role in mediating host resistance against influenza virus replication. For example, *IRF-7* knockdown resulted in enhanced PR8 influenza virus production in A549 cells and MDCK cells [29]. This study also found high basal expression of key antiviral genes namely *STING*, *ISG15* and *Mx1* in BEAS-2B cells relative to NHBE and A549 cells. *ISG15* is one of the most highly induced IFN-induced proteins and inhibits replication of a wide range of viruses including IAVs [30]. High basal expression of Mx is also a key marker of virus resistance in cells. For example, vesicular stomatitis virus (VSV) resistant human pancreatic ductal adenocarcinoma (PDA) cell lines displayed high basal expression of ISGs *MxA* and 2′,5′-oligoadenylate synthetase (*OAS*) in comparison with VSV susceptible PDA cells [31]. *STING* plays an important role in host defence against a range of intra-cellular pathogens and acts as a cytosolic DNA sensor [32]. The collective evidence suggests that resistance of BEAS-2B cells to IAV infection is mediated by high basal expression of antiviral genes and their subsequent strong induction (by 24 h) in response to infection. Furthermore, high basal expression of cytosolic DNA sensor *STING* in BEAS-2B cells raises the possibility that these cells could also be resistant to DNA viruses. Hence, it is prudent to determine the susceptibility of BEAS-2B cells to other viruses to assess its usefulness as an *in vitro* model for the study of virus cytopathogenesis.

Distinct profiles of antiviral gene responses following IAV infection were also noticed among the three respiratory epithelial cells (BEAS-2B, NHBE and A549 cells). In particular, the regulation of antiviral genes at 10 h of virus infection was highly variable among the cell types. Notably, A549 cells showed significantly higher expression of *IFN-β* and *IL-6* expression than BEAS-2B and NHBE cells at 10 h of infection. This could be due to higher levels of IAV replication in A549 cells compared with other cells. IL-6 is a vital innate immune cytokine in the protection against severe influenza A infection [33]. *IL-6* induction was highest in A549 cells at 10 h of infection, while it was highest at 24 h of infection in BEAS-2B and NHBE cells. While we do not know the significance of the distinct cytokine expression profiles among the three cells, clearly there were inherent differences in IAV cytopathogenesis in these cells. It is well recognised that inhibition of JAK/STAT signalling results in reduced ISG expression [31]. We showed that JAK/STAT inhibition by P6 treatment reversed the antiviral state of BEAS-2B cells as evident by the lower mRNA level of ISGs. Enhanced susceptibility of P6 treated BEAS-2B cells to IAV infection is evident from increased viral M gene RNA detection and increased production of infectious progeny virus.

In conclusion, comparison of IAV replication in three human respiratory epithelial cell types along with MDCK cells showed BEAS-2B cells to be highly resistant to avian H2N3 and human H1N1 IAV infection. Resistance of BEAS-2B cells to H1N1 virus was mediated by high basal expression of antiviral genes namely *IRF-7*, *STING*, *ISG15* and *Mx1*. JAK/STAT inhibition by P6 treatment reversed the antiviral state of BEAS-2B cells and resulted in increased H1N1 virus replication. Studies continue to make inferences on IAV pathogenesis based on results of infected BEAS-2B cells [27], [34], [35]. However, it is clear from our data that IAV replication and host response in BEAS-2B cells are different from primary NHBE cells. Our findings warrant evaluation of BEAS-2B cells for suitability for use with other human respiratory viruses. This study highlights the importance of using appropriate cell culture models that closely reflect viral pathogenesis in a given host species to make valid inferences.
